# COVID-19 and breast cancer: may the microbiome be the issue?

**DOI:** 10.2217/fon-2020-0764

**Published:** 2020-11-27

**Authors:** Angioletta Lasagna, Valentina Zuccaro, Elisa Ferraris, Marta Corbella, Raffaele Bruno, Paolo Pedrazzoli

**Affiliations:** ^1^Medical Oncology Unit, Fondazione IRCCS Policlinico San Matteo, Viale Camillo Golgi 19, 27100, Pavia, Italy; ^2^Division of Infectious Diseases I, Fondazione IRCCS Policlinico San Matteo, Viale Camillo Golgi 19, 27100, Pavia, Italy; ^3^Department of Microbiology & Virology, Fondazione IRCCS Policlinico San Matteo, Viale Camillo Golgi 19, 27100, Pavia, Italy; ^4^Department of Clinical Surgical Diagnostic & Pediatric Sciences, University of Pavia, Viale Camillo Golgi 19, 27100, Pavia, Italy; ^5^Department of Internal Medicine & Medical Therapy, University of Pavia, Viale Camillo Golgi 19, 27100, Pavia, Italy

**Keywords:** breast cancer, COVID-19, estrobolome, estrogen, gut microbiota, microbiome, SARS-CoV-2, X chromosome

The impact of the COVID-19 pandemic on cancer patients represents a major issue for the oncologist. Although many epidemiological data have been collected so far, understanding some aspects of the relationship between some types of cancer and COVID-19 remains a challenge. In this commentary we hypothesize a relationship between breast cancer (BC), gut microbiota (GM) and COVID-19 by reviewing existing data to predict possible impact on clinical practice.

## COVID-19 & cancer: are some types of cancer more susceptible?

Cancer is a common comorbidity in COVID-19, and infected cancer patients have much more severe illness and a nearly threefold increase in the death rate compared with COVID-19 patients without cancer [1]. It has now been established that SARS-CoV-2 uses the ACE2 receptor for entry and TMPRSS2 for S protein priming [2]. A recent meta-analysis reported that patients with lung cancer and colorectal cancer are more susceptible to SARS-CoV-2 infection: supporting bioinformatic data shows an increased level of mRNA expression of both *ACE2* and *TMPRSS2* in these cancer types [3]. However, the small sample size and the inclusion of only seven types of cancer led us to interpret the conclusions with some caution. So far the available data has not allowed us to define whether there is a cancer type more susceptible to COVID-19 because the case series have a wide heterogeneity and data have not been disaggregated and analyzed by sex. Indeed, emerging global observations suggest that women are at lower risk of both infection and mortality from COVID-19, as compared with men [4].

## Sex differences in COVID-19: the role of estrogens

In general, women are less susceptible to COVID-19 based on a different innate immunity, steroid hormones and factors related to sex chromosomes [4]. The influence of stochastic X chromosome inactivation is one of the possible explanations, because ACE2 is encoded by the *ACE2* gene located on the X chromosome.

Male cells always express a single *ACE2* allele, while females have a potentially more efficient form of ACE2 receptor which would be present, on average, in only half of cells. Although this mechanism may limit susceptibility to infection with the SARS-CoV-2 virus giving women a relative resistance [5], it is probably not enough to explain women's differing susceptibility to infection. In a recent review of mechanistic differences in the expression and activity of ACE2, the authors reported that ACE2 activity in a mouse model was greater in the male kidney and this sex difference was driven by estradiol reducing ACE2 activity regardless of the sex chromosome complement [6].

It is well known that sex hormones are able to affect the innate and adaptive immunological response: the androgens have anti-inflammatory effects and the estrogens have both pro- and anti-inflammatory ones [7]. In particular, as summarized in a detailed and inspiring review about biological interactions and molecular links between prostate cancer and SARS-CoV-2, estrogens suppress the levels of pro-inflammatory IL-6 by directly altering CD16 expression, and can influence the levels of natural killer cells [8]. The key role of estrogens has been hypothesized in a cross-sectional study carried out in Wuhan: Ding and colleagues analyzed the correlation between menstrual status, female hormones and cytokines related to immunity and inflammation, and the severity and clinical outcomes in female COVID-19 patients aged <60 years. Estradiol showed a negative correlation with severity of infection and the authors found that menopause is an independent risk factor for COVID-19 patients [9]. The role of estrogen appears to be of emerging importance, but how does it influence hormone-dependent cancers specifically?

## GM, estrogens, estrobolome & BC

The relevance of this question is highlighted by an analysis of the results of a cohort study reporting data from the COVID-19 and Cancer Consortium Registry database that surprisingly showed BC (21%) and prostate cancer (16%), two strongly hormone-dependent tumors, to be the most common types of cancer among 1018 cases of COVID-19 accrued in March–April 2020 [10]. This finding supports the hypothesis that GM composition may represent a factor able to explain the association.

Accumulating data in the literature show that the microbial composition of GM is subject to sex differences in prevalence of species and function, in part driven by sex hormones: estrogen levels in men and postmenopausal women directly correlate with GM richness and diversity, whereas there is no correlation in premenopausal women [11]. Moreover, the GM seems capable of modulating serum estrogen levels and promoting the proliferation of certain species of bacteria; Plottel and Blaser defined the aggregate of enteric bacterial genes whose products are capable of metabolizing estrogens as the ‘estrobolome’ [12]. Hepatic conjugation of estrogen leads to the secretion of both conjugated estrogen and conjugated estrogen metabolites into the GI tract where they are transformed into free forms of these molecules by β-glucuronidases, glucosidases and hydroxysteroid dehydrogenases of bacterial origin [12]. If the estrobolome is rich in bacteria with higher deconjugative and hydroxylating enzymatic activity, this leads to greater relative levels of circulating free estrogens [12]. In the human GI tract, the most important β-glucuronidase-encoding genes are named GUS genes; approximately 112 novel GUSs have been identified and clustered into six classes expressed in four bacterial phyla, namely *Bacteroidetes*, *Firmicutes*, *Verrucomicrobia* and *Proteobacteria* [13]. Among these, *Bacteroidetes* presents the highest abundance and diversity of GUS enzymes [13].

BC is the most frequent tumor in women, and accumulating data indicate an increasing incidence rate: in 2018, the estimated age-adjusted annual incidence of BC in 28 EU countries was 144.9/100,000 and the mortality 32.9/100,000 [14]. BC has many risk factors, but several studies have analyzed the complex relationship between BC and the estrogen-dependent functions of the gastrointestinal microbiome [15]. In a study published in 2018, BC cases had significant estrogen-independent associations with the IgA-positive and IgA-negative GM compared with controls. These findings suggest that BC risk may be influenced through enterohepatic cycling of estrogens by the IgA-negative microbiota and through immune-mediated pathways by the IgA-positive microbiota [16]. Indeed, when evaluating the composition of the GM among BC patients with different clinical characteristics, the absolute numbers of *Bifidobacterium* and *Blautia*, and proportions of *Faecalibacterium prausnitzii* and *Blautia*, varied according to the clinical stage of cancer [17]. An increase in the ratio of *Firmicutes* to *Bacteroide*s has been observed in BC patients compared with BC-free individuals [18].

If GM alterations affect the estrobolome activity in BC patients, what do we know about bacterial diversity and COVID-19? Intriguingly, a recent paper reported an association between gut *Firmicutes* bacteria and COVID-19 severity. The main bacterial species that show a negative correlation with COVID-19 severity are *Alistipes onderdonkii* and *F. prausnitzii*; *Bacteroides* species display a potential protective role against SARS-CoV-2 infection by hampering host entry through ACE2 [19]. Despite the modest sample size of this exploratory study (which used fecal samples from 15 patients with COVID-19, 6 subjects with community-acquired pneumonia and 15 healthy individuals), these findings altogether suggest that an individual's gut microbiome configuration may affect their susceptibility and response to SARS-CoV-2 infection [19].

## BC & susceptibility to COVID-19: is the estrobolome the balance needle?

In light of all this evidence, two questions may arise: what is the relationship between COVID-19 and BC, and could the estrobolome affect it? Unfortunately, we do not yet have sufficient data to achieve a definitive conclusion, but we could formulate interesting hypotheses and try to suggest methods to investigate them.

Although we have no information about the real incidence of the infection in BC patients, or about which subtype (luminal A-like, luminal B-like, triple negative and HER2 positive) of BC is most involved, we know that in BC patients, estrogen is produced primarily by adipose tissue and via the aromatization of androgen precursors; aromatase inhibitors, one of the principal therapeutic approaches for estrogen receptor-positive BC in postmenopausal women, potently inhibit aromatase activity and suppress estrogen levels in plasma and tissue [20]. GM composition influences various aspects of hormone regulation. This process upregulates circulating levels of steroid hormones and cytokines that are known to increase the risk and progression of BC [21]. Dysbiosis has been found to be associated with postmenopausal but not premenopausal BC [22] but the nature of the interactions between aromatase inhibitors and GM has not been completely established. So far, data about the relationship between aromatase inhibitors and GM composition are lacking, although the modulation of the GM by selective estrogen receptor modulators (tamoxifen and raloxifen) is well known [23]. We must also deal with many confounding factors, such as BMI, age and antibiotic intake; all of these can alter GM and increase the complexity of this intricate puzzle [15]. Moreover, BMI and aging are well-known risk factors for COVID-19 [24].

We can speculate that the estrobolome plays a role in modifying susceptibility to COVID-19 by modulating estrogen levels. Establishing a relationship between GM alterations linked to BC and COVID-19 is not only a stylistic exercise but may be important because of the high number of cases and the need for an individualized approach to identify patients at risk. To evaluate this link between BC and susceptibility of COVID-19, we suggest three strategies:

- The collection and storage of biological samples (stool and plasma) to assess the links between GM composition and estrobolome activity by cross-sectional studies;
- The creation of a large database collecting the characteristics of BC with regard to ongoing therapies, especially among estrogen receptor-positive subtypes treated with endocrine therapy, and the incidence rate of COVID-19;
- The analysis of database with machine learning algorithms to find any correlations existing among estrobolome, BC and COVID-19.

## Conclusion

Based on available data the paradigm that BC patients may be protected against COVID-19 by increased estrogen levels is now under discussion and many questions arise, including whether endocrine therapy can interfere with the estrobolome and can make patients more susceptible to COVID-19 infection.

While COVID-19 spreads as a pandemic all over the world, oncologists should explore all strategies, however visionary, to protect their patients. Investigating the link between BC, the estrobolome and COVID-19, identifying a population of patients with hormone-sensitive cancer as being at increased risk for COVID-19, and setting strategies to prevent it by modifying their GM, may be a fascinating challenge for oncologists in the near future. What is certain is that we badly need more robust epidemiological, preclinical and clinical data to guide us in our daily practice.
